# Molecular detection of avian parasites in Australian mosquitoes (Culicidae)

**DOI:** 10.1093/jme/tjaf142

**Published:** 2025-10-07

**Authors:** Ashleigh M Peck, Alan Lymbery, Siobhon Egan, Amanda Ash

**Affiliations:** School of Medical, Molecular and Forensic Sciences, Murdoch University, Murdoch, Western Australia, Australia; School of Environmental and Conservation Sciences, Murdoch University, Murdoch, Western Australia, Australia; Centre for Sustainable Aquatic Ecosystems, Harry Butler Institute, Murdoch University, Murdoch, Western Australia, Australia; Centre for Computational and Systems Medicine, Murdoch University, Murdoch, Western Australia, Australia; Centre for Biosecurity and One Health, Murdoch University, Murdoch, Western Australia, Australia; School of Medical, Molecular and Forensic Sciences, Murdoch University, Murdoch, Western Australia, Australia; Centre for Biosecurity and One Health, Murdoch University, Murdoch, Western Australia, Australia

**Keywords:** *Culex*, *Plasmodium*, *Haemoproteus*, *xenomonitoring*

## Abstract

Mosquitoes (Culicidae) are the most important vectors of human and animal diseases globally, making them valuable tools for the molecular surveillance of blood-borne pathogens. By screening mosquito populations, we can evaluate local disease prevalence and ascertain which vector species are involved in local transmission cycles. This study presents the first targeted mosquito-based surveillance of blood parasites in Western Australia. Over a 2-year surveillance program in Perth, Western Australia, 3,288 mosquitoes from 12 species across 5 genera were collected and screened in 461 pools. Parasite prevalence and diversity were evaluated using polymerase chain reaction screening of the Haemosporida cytochrome *b* gene region, and the *Dirofilaria* 12S rDNA gene region. Haemosporida were detected in 3.9% of mosquito pools, with 72.2% of positives found in *Culex* species pools. Avian Haemosporida comprised 83.3% of the total detections. Known avian Haemosporida lineages detected included 1 *Haemoproteus* (*H*. *zosteropis*) and 2 *Plasmodium* (BELL01 and MYNA02). Three novel lineages, *Plasmodium* CULPER01-03, were identified. *Plasmodium falciparum* was identified in 2 pools, and no *Dirofilaria* were detected. These findings indicate that Perth harbors a diverse range of avian Haemosporida, which may be regionally specific, as all lineages detected have only been identified in the Oceania region. The predominance of positive detections in the *Culex pipiens* species complex supports their role as the primary vectors of avian *Plasmodium*. This study highlights the utility of mosquito surveillance for monitoring blood-borne parasites and contributes new insight into parasite diversity and vector associations in Australia.

## Introduction

Mosquitoes are important vectors of human and animal parasites, with the most important parasites being protozoans, in the order Haemosporida, and filarial nematodes (WHO 2020; Mullen and Durden 2018, Cromwell et al. 2020, Genchi and Kramer 2020). Successful transmission between mosquito vectors and vertebrate hosts relies on the prevalence and interaction of competent vectors, susceptible hosts, and parasites (Keesing et al. 2006, Brugueras et al. 2020). A mosquito’s competence as a vector depends on the parasite’s ability to evade internal transmission barriers, which prevent pathogen dissemination and transmission. A parasite’s evasion has evolved to be highly vector species-specific; hence, vector competence varies between mosquito species (Kramer et al. 1981, Romoser et al. 2005, Turell et al. 2013, Franz et al. 2015, Martínez-de la Puente et al. 2018). Similarly, parasites co-evolve with their host to reduce infection cost. Essentially, infections are efficiently maintained between competent vectors and susceptible vertebrate hosts (Ebert and Fields 2020). This results in a dynamic system shaped by the biodiversity of each component (vector, host, and parasite), which varies over time and space.

Mosquitoes are important vectors for filarial nematodes, which infect humans and animals. Human filarial nematodes include *Brugia malayi*, *B*. *timori*, and *Wuchereria bancrofti*, which cause human lymphatic filariasis and are limited to Africa, Asia, and the Americas (World Health Organization 2013, Cromwell et al. 2020). In contrast, the canine filarial nematode, *Dirofilaria immitis*, commonly known as the dog heartworm, is globally distributed (Genchi and Kramer 2020). *Dirofilaria immitis* can be lethal to canines when adult worms reside in the pulmonary arteries, causing congestive heart failure (McCall et al. 2008). This parasite is strongly associated with warmer climates, with prevalence across landscapes demonstrating increasing gradients in the United States and Europe from north to south, and in Australia from south to north (Brown et al. 2012, Morchón et al. 2012, Wang et al. 2014, Atkinson et al. 2025). Despite observed gradients, treatment programs in the United States and Australia are prescribed based on a homogeneous risk, with blanket treatment initiatives nationwide (American Heartworm Society 2024, Atkinson et al. 2025). However, the data on *D*. *immitis* prevalence in Australia’s southwest are extremely limited, with the last study published in 1989 (Edwards et al. 1989).

Parasites of the order Haemosporida, including those in the genera *Plasmodium* and *Haemoproteus*, are protozoans suspected to be transmitted by mosquitoes and biting midges, respectively, and parasitise humans and other animals, including birds, reptiles, rodents, and non-human primates (Mullen and Durden 2018). The competent mosquito species for *Plasmodium* spp. differs depending on its intermediate host species, notably, human malaria is primarily transmitted by *Anopheles* spp., whilst avian malaria is suspected to be transmitted by species within the *Culex pipiens* complex (Žiegytė et al. 2014, Schoener et al. 2017, Wanger et al. 2017, Köchling et al. 2023, Garrigós et al. 2024).

Avian Haemosporida have been detected worldwide, except in Antarctica, with fewer detections in the Oceania region (Clark et al. 2014). On average, 12.8% of avian populations are infected with a *Plasmodium* species. However, infection probability varies due to regionally specific landscape variation and the biodiversity of host and vector species, influencing vector-host interactions (Fecchio et al. 2021). Avian Haemosporida diversity exhibits geographical patterns, with diversity trends mirroring those of local avian host populations (Clark et al. 2014).

To date, a high diversity of avian Haemosporida has been identified, as molecular methods have uncovered a more extensive species richness than that inferred by morphology (Valkiūnas 2011). Molecular screening of the cytochrome *b* (cyt *b*) gene region has revealed 3,475 unique lineages. Only 220 of these species have been morphologically described to species level, as catalogued in the MalAvi database, a unified repository for avian Haemosporida cyt* b* sequences (http://130.235.244.92/Malavi/, Accessed in April 2025, Bensch et al. 2009). Avian Haemosporida has high lineage numbers, as even a single-nucleotide polymorphism (SNP) variation in the cyt *b* gene region is recognized as a novel lineage (Beadell et al. 2004, Reullier et al. 2006, Lachish et al. 2013, Bensch et al. 2009). However, a sequence divergence of 5% (approximately 25 nucleotides) or more correlates well with morphologically distinct species (Hellgren et al. 2007).

Each lineage may exhibit differing host and geographical range (Beadell et al. 2004, Bensch et al. 2000, Reullier et al. 2006, Lachish et al. 2013, Santiago-Alarcon et al. 2019). For example, the avian *Plasmodium* lineage BELL01 appears to be geographically restricted to the Oceania region and has only been detected in 3 avian families (Callaeidae, Meliphagidae, Sturnidae) (Baillie and Brunton 2011, Ewen et al. 2012, Clark et al. 2015). In contrast, *P*. *homocircumflexum* (lineage COLL4) has a wide geographical distribution across Asia, North and South America, Africa, and Europe, and has been identified in 12 different avian families with pathogenicity varying between species (Kulma et al. 2013, Levin et al. 2013, Kulma et al. 2014, Garamszegi et al. 2015, Palinauskas et al. 2015, Ilgūnas et al. 2016, Szöllösi et al. 2016, Jones et al. 2018, Musa et al. 2019, DeBrock et al. 2021, Doussang et al. 2021, Musa et al. 2022).

The importance of understanding the geographical distribution and host-lineage interactions of avian Haemosporida has been highlighted through significant host mortality events. Postmortem results of severe Haemosporida infections have demonstrated damage to the spleen, heart, and liver leading to death of their avian host (Ilgūnas et al. 2016), while asymptomatic chronic infections can lead to reduction in lifespan and reproduction success (Asghar et al. 2015).

In the early 1900s, the rapid spread of *P*. *relictum* (lineage GRW04) led to the mass extinction of 23 native birds in Hawaii. Hawaii’s mass extinction was triggered by the introduction of the competent and invasive mosquito *Cx*. *quinquefasciatus*, establishing the transmission of *P*. *relictum* to immunologically naïve birds (van Riper et al. 1986, Martínez-de la Puente et al. 2018). This mass extinction event has raised concern for the conservation of native avian populations and sparked the surveillance of avian Haemosporida worldwide.

Whilst it is widely accepted that immunologically naïve avian populations are highly susceptible to severe infections (Zehtindjiev et al. 2013, Palinauskas et al. 2015, Ilgūnas et al. 2016), similar severe outcomes have been reported in adapted avian populations (Loiseau et al. 2011, Ebert and Fields 2020). Essentially, pathogenic outcomes depend on the host–parasite combination, highlighting the system’s complexity. Therefore, understanding host-lineage interactions is an essential aspect of avian conservation efforts.

Currently, there is limited information on the diversity of avian Haemosporida in Australia. A geographically distinct diversity of avian Haemosporida is expected given Australia’s geographical isolation, unique avian population, and prevalence of competent vectors (Fecchio et al. 2021). Out of the 3,475 identified lineages, only 135 have been found in Australia, with 80% of these detected exclusively in the country (MalAvi database: Checked 28 April 2025). However, this is not a comprehensive list of all lineages, as surveillance has only been conducted on a limited number of avian species collected in eastern and northern regions of Australia (Adlard et al. 2004, Beadell et al. 2004, Ishtiaq et al. 2006, Zamora-Vilchis et al. 2012, Balasubramaniam et al. 2013, Laurance et al. 2013, Clark et al. 2015, Goulding et al. 2016, Verwey et al. 2018, Eastwood et al. 2019). In particular, the southwest region of Australia, a recognized biodiversity hotspot with a very high degree of endemism (Myers et al. 2000), remains underrepresented in the literature. The south of Western Australia is also home to 90% of the state’s population, with 80% residing in the capital city, Perth. Perth is notably isolated, with the next closest capital city, Adelaide, located 2,104 km away. Such isolation makes Perth a distinctive location for the surveillance of mosquito-borne parasites. This study presents the results of a 2-year mosquito surveillance program in Perth, Western Australia. Our aim was to characterise the diversity of Haemosporida and filarial parasites and evaluate the potential vector roles of local mosquito species in their transmission.

## Materials and Methods

### Study Site and Mosquito Collection

From October 2022 to August 2024, 10 locations were sampled across Perth metropolitan areas (Coordinates in Supplementary Table S1). Adult mosquitoes were collected using dry-ice-baited CDC traps emitting CO_2_. Traps were set 14 times over 2 yr to observe changes in seasonal mosquito and parasite diversity. All traps were baited on the same day and ran overnight for 16 to 20 h. Trapping time varied across seasons, as the last trap in the circuit was set 1 h before sunset, and the first trap was collected 1 h after sunrise. Mosquitoes were knocked down by placing the catch container in an esky with dry ice. All mosquitoes were stored at −80 °C until further procedures.

All mosquitoes were morphologically identified on a refrigerated table with a stereo microscope. Species were identified using Australian mosquito identification keys (Lee et al. 1980, Liehne et al. 1991). All female mosquitoes were pooled in an Eppendorf tube according to species, location and collection date, ranging from 1 to 30 individuals; however, for the most prevalent species, *Ae*. *notoscriptus*, the maximum was up to 60.

### DNA Extraction and Amplification

Each Eppendorf tube was placed in liquid nitrogen to freeze mosquitoes and then pulverized with a mortar. DNA was extracted using a DNeasy Blood and Tissue Kit (QIAGEN), following the standard protocol with a minor addition to the sample digest: a 1-min centrifugation at 2,000 rpm after 100% ethanol was added to pellet the undigested exoskeleton.

Polymerase chain reaction (PCR) protocols designed to amplify DNA from filarial nematodes and Haemosporida species were conducted on all mosquito pools. A ∼450bp segment of the conserved 12S rDNA gene region of filarial nematodes, including *Dirofilaria* spp., was amplified using PCR primers 12SF (5′-GTTCCAGAATAATCGGCTA-3′) and 12SR (5′-ATTGACGGATG(AG)TTTGTACC-3′) (Casiraghi et al. 2004). Reactions were carried out in 20 µl volumes each containing 2 µl of DNA, 1.1 units of Taq polymerase (ThermoFisher Scientific), 0.4 mM dNTPs (ThermoFisher Scientific), 5 mM MgCl_2_, 1.25× buffer, and 0.5 µM of each primer. Thermocycler conditions were as follows: 95 °C for 3 min, followed by 40 cycles of 95 °C for 30 s, 55 °C for 45 s, and 72 °C for 90 s, and a final extension at 72 °C for 8 min. Each reaction contained a PCR-grade water negative control and a filarioid-positive sample for *Breinlia jittapalapongi* (Asian house rat filarial nematode).

DNA from Haemosporida within the genera *Plasmodium* and *Haemoproteus* was amplified using a nested PCR for the cytochrome *b* gene region (Bensch et al. 2000). Primary PCR primers HaemNF (5′-CATATATTAAGAGAATTATG GAG-3′) and HaemNR2 (5′-AGAGGTGTAGCATATCTATC TAC-3′) amplified a ∼580 bp fragment and secondary PCR primers HaemF (5′-ATGGTGCTTCGATATATGC ATG-3′) and HaemR2 (5′-GCATTATCTGGATGTGATAA TGGT-3′) amplified a ∼520 bp fragment. Reactions were carried out in 25 µl volumes each containing 2 µl DNA template for the primary reaction or 2 µl of the primary amplicon for the secondary reaction, 0.8 units of Taq polymerase (ThermoFisher Scientific), 0.48 mM dNTPs (ThermoFisher Scientific), 3 mM MgCl_2_, 1× Buffer, and 0.6 µM of each primer. Primary reaction PCR thermocycler conditions were as follows: 95°C for 3 min, followed by 30 cycles of 95 °C for 30 s, 50 °C for 30 s, and 72 °C for 45 s, and a final extension at 72 °C for 10 min. The secondary reaction PCR thermocycler conditions were the same but included 35 cycles instead of 30 cycles. Each reaction contained a PCR-grade water-negative control and a *P*. *falciparum*-positive blood sample.

PCR products were separated by gel electrophoresis in a 1.5% agarose gel stained with SYBR Safe (ThermoFisher). Amplified products were purified using the Agencourt AMPure XP method and sent for sequencing by the State Agricultural Biotechnology Centre, Australia. Sequencing chromatograms were edited and aligned using Geneious Primer version 2021.0.3 (http://www.geneious.com) (Kearse et al. 2012). Sequence alignments were identified using BLAST search tools through the NCBI (https://blast.ncbi.nlm.nih.gov/) and MalAvi databases (http://130.235.244.92/Malavi/, Bensch et al. 2009).

Haemosporida sequences with 100% homology were classified as the aligned lineage from the MalAvi database. Sequences with one or more nucleotide differences from published lineages were classified as a novel lineage (Beadell et al. 2004, Waldenström et al. 2004, DeBrock et al. 2021). All detected lineages were deposited in the MalAvi database and GenBank (Accession no. PX317471- PX317488).

The prevalence of parasite infection was calculated using the maximum likelihood estimate of the pooled prevalence, implemented with PoolTools V0.1.5 (McLure et al. 2021; available at: https://poolbox.shinyapps.io/PoolTools/).

The phylogenetic analysis included reference sequences with the closest alignments to positive sequences from the MalAvi database to demonstrate lineage diversity. Phylogenetic analysis was performed using Bayesian inference run by MrBayes v3.2.7 (Ronquist and Huelsenbeck 2003). The tree shows nodes with a posterior probability of 0.5 or more. The consensus tree was displayed in FigTree v.1.4.4 (http://tree.bio.ed.ac.uk/software/figtree/).

The evolutionary history of Haemosporida lineages was also inferred using the maximum likelihood method, selecting the nucleotide substitution model that best fit this DNA data using the Akaike information criterion and Bayesian information criterion in MEGA11 (Tamura et al. 2021). Analysis determined that the GTR + I model best fit the data.

## Results

This study screened 3,288 mosquitoes from 12 different species within the genera *Aedes* (4 species), *Anopheles* (1 species), *Coquillettidia* (1 species), *Culex* (5 species), and *Culiseta* (1 species). *Aedes notoscriptus* made up 53.86% of all mosquitoes screened. Mosquitoes were separated into 461 mosquito pools from *Aedes* spp. (*N* = 219 pools), *Culex* spp. (*N* = 200), *Culiseta atra* (*N* = 9), *Coquillettidia* sp. nr. *linealis* (*N* = 18), and *Anopheles annulipes* (*N* = 15) (Table 1).

**Table 1.** The number of mosquitoes sampled and the Haemosporida lineages detected within the Perth metropolitan region, Western Australia

Genus	Species	Number of samples no. (%)	Total number of mosquito pools no. (%)	Total positive pools no. (%)	Haemosporidia							
					*Plasmodium*						*Haemosporida*	*Dirofilaria*
					Lineage: CULPER			BELL01	MYNA02	*falciparum*	*zosteropis*	spp.
					1	2	3					
***Aedes***	*Alboannulatus*	144 (4.38)	53 (11.50)	2 (11.11)		2						
	*camptorhynchus*	160 (4.87)	26 (5.64)	0								
	*notoscriptus*	1772 (53.86)	122 (26.46)	1 (5.56)			1					
	*vigilax*	169 (5.14)	18 (3.90)	0								
***Anopheles***	*annulipes*	40 (1.22)	15 (3.25)	0								
***Coquillettidia***	sp*.* nr*. linealis*	124 (3.77)	18 (3.90)	0								
***Culex***	*Annulirostris*	204 (6.20)	35 (7.59)	2 (11.11)						2		
	*australicus*	198 (6.02)	47 (10.20)	5 (27.78)	1	2		1	1			
	*Globocoxitus*	145 (4.41)	35 (7.59)	4 (22.22)	1	2		1				
	*molestus*	57 (1.73)	26 (5.64)	0								
	*quinquefasciatus*	259 (7.88)	57 (12.36)	2 (11.11)		1		1				
***Culiseta***	*atra*	16 (0.48)	9 (1.95)	2 (11.11)	1						1	
**Total**		**3288**	**461**	**18**	3	7	1	3	1	2	1	None detected

Of the 461 mosquito pools, 18 were positive for Haemosporida parasites (3.9%). A single *Haemoproteus* lineage was detected in a *Culiseta atra* pool. *Plasmodium* spp. were the most prevalent (94.44%), with the majority aligning with avian *Plasmodium* lineages (88.22%). *Culiseta atra* had the highest percentage of positive mosquito pools, at 22.22%; however, 73.33% (11/15) of the pools were from the *Culex pipiens* complex. The highest estimated prevalence of Haemosporida in mosquitoes was found in *Culiseta atra* (14.6%, 95% CI 2.56% to 39.42%), followed by *Cx*. *globocoxitus* (2.86%, 95% CI 0.9% to 6.52%) and *Cx. australicus* (2.76%, 95% CI 1.0% to 5.85%) (Table 2). *Aedes* spp. also tested positive for Haemosporida, with 2 positive pools for *Ae*. *alboannulatus* and one for *Ae*. *notoscriptus* (Table 1). No Haemosporida were detected in pools of the genera *Anopheles* or *Coquillettidia.* No *Dirofilaria* spp. were detected in this study.

**Table 2.** Maximum likelihood estimates of individual Haemosporida prevalence from pooled mosquito samples

Haemosporida	Mosquito species	Number of mosquitoes	Number of pools	Number of positive pools	Prevalence (maximum likelihood estimate)	95% confidence interval
**All**	*Ae*. *Alboannulatus*	144	53	2	0.014	0.0023–0.0427
	*Ae*. *camptorhynchus*	160	26	0	0	0–0.0119
	*Ae*. *notoscriptus*	1772	122	2	0.0006	0–0.0025
	*Ae*. *vigilax*	169	18	0	0	0–0.0113
	*An*. *annulipes*	40	15	0	0	0–0.0469
	*Coquilletidia* sp. nr. *linealis*	124	18	0	0	0–0.0154
	*Cx*. *annulirostris*	204	35	2	0.0107	0.0018–0.0327
	*Cx*. *australicus*	198	47	5	0.0276	0.01–0.0585
	Cx. *globocoxitus*	145	35	4	0.0286	0.009–0.0652
	*Cx*. *molestus*	57	26	0	0	0–0.0331
	*Cx*. *quinquefasciatus*	259	57	2	0.0081	0.0014–0.025
	*Culiseta atra*	16	9	2	0.1461	0.0256–0.3942
**BELL01**	*Cx*. *australicus*	198	47	1	0.0051	0.0003–0.0224
	Cx. *globocoxitus*	145	35	1	0.007	0.0004–0.0307
	*Cx*. *quinquefasciatus*	259	57	1	0.0039	0.0002–0.0172
**MYNA02**	*Cx*. *australicus*	198	47	1	0.0052	0.0003–0.0228
**CULPER01**	*Cx*. *australicus*	198	47	1	0.0051	0.0003–0.0221
	Cx. *globocoxitus*	145	35	1	0.007	0.0004–0.0303
	*Culiseta atra*	16	9	1	0.0625	0.0037–0.2475
**CULPER02**	*Ae*. *alboannulatus*	144	53	2	0.014	0.0023–0.0427
	*Cx*. *australicus*	198	47	2	0.0104	0.0017–0.0318
	Cx. *globocoxitus*	145	35	2	0.0138	0.0023–0.0421
	*Cx*. *quinquefasciatus*	259	57	1	0.004	0.0002–0.0175
**CULPER03**	*Ae*. *notoscriptus*	1772	121	1	0.0006	0–0.0025
***H*. *zosteropis***	*Culiseta atra*	16	9	1	0.0722	0.0042–0.2858
***P*. *falciparum***	*Cx*. *annulirostris*	204	35	2	0.0107	0.0018–0.0327

**Fig. 1.**
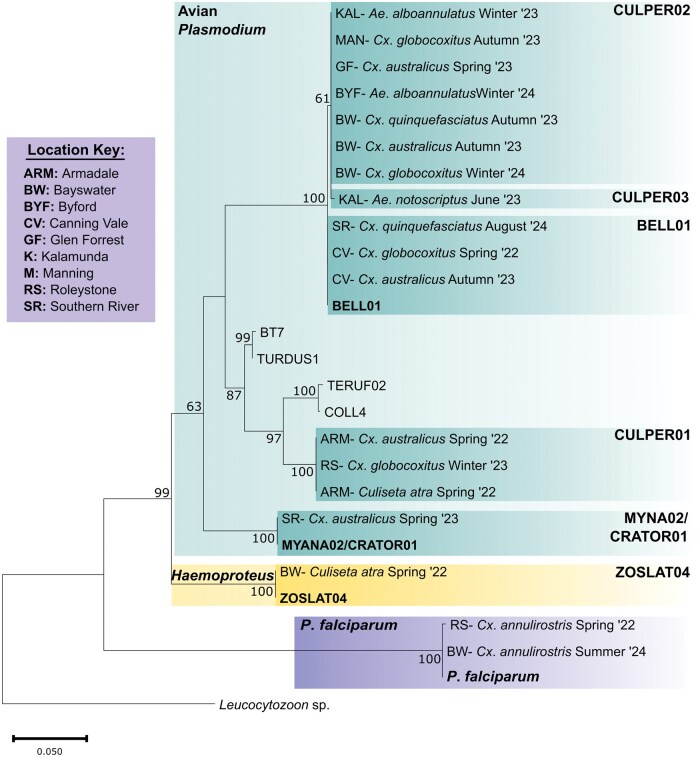
Maximum Likelihood tree of the Haemosporida lineage identified in the Perth mosquito population and the closest genetic alignments. The sample naming convention is an abbreviation of the location, followed by the mosquito species and the sampling date. Lineage names from MalAvi are bolded. Such lineage names have a 100% homology with the detected lineages in Perth, Western Australia.

Seven Haemosporida lineages were identified, and phylogenetic relationships were inferred using a maximum likelihood tree (Fig. 1), with a similar topography obtained for the Bayesian tree (Supplementary Fig. S1).

*Plasmodium falciparum* (100% homology to GenBank KY923542) was identified in 2 pools of *Cx. annulirostris* (2/18) at 2 sites (Bayswater and Roleystone). Three previously identified avian Haemosporida lineages were detected: one *Haemoproteus* lineage, *H*. *zosteropis*, lineage ZOSLAT04 (1/18, 100% homology to GenBank JX021550) identified in a single *Culiseta atra* pool; and 2 *Plasmodium* lineages, BELL01 (3/18, 100% homology to GenBank JQ905572), and MYNA02 (1/18, 100% homology to GenBank KT777733) identified within *Culex* spp. pools (Table 3).

**Table 3.** Information on the 3 previously identified avian *Plasmodium* and *Haemoproteus* lineages, which were detected in mosquitoes in this study

Genus	Lineage/species (Accession no.)	No. detected	Mosquito species detected in (no. positive pools)	Host family previously identified	Previous regions identified	References
*Plasmodium*	BELL01 (JQ905572)	3	*Cx*. *australicus* (1) *Cx*. *globocoxitus* (1) *Cx*. *quinquefasciatus* (1)	Callaeidae, **Meliphagidae**, Sturnidae	Oceania (Australia- NSW, New Zealand)	Baillie and Brunton 2011, Ewen et al. 2012, Clark et al. 2015
	MYNA02 (KT777733)	1	*Cx*. *australicus* (1)	**Artamidae**, Sturnidae	Oceania (Australia- NSW, New Zealand)	Clark et al. 2015, Goulding et al. 2016, Schoener et al. 2020
*Haemoproteus*	*zosteropis* (JX021550)	1	*Culiseta atra* (1)	**Zosteropidae**	Oceania (Australia- QLD, New Caledonia)	Zamora-Vilchis et al. 2012

The bolded families indicate that species within that family reside in Perth, Western Australia. QLD, Queensland; NSW, New South Wales.

Information includes the avian families and regions from which each lineage has been reported.

Three novel avian *Plasmodium* lineages were discovered (CULPER01-03) using the criteria that one or more nucleotide differences from published lineages are classified as a novel lineage (Beadell et al. 2004, Waldenström et al. 2004, DeBrock et al. 2021). The novel lineage CULPER01 was identified in 3 mosquito pools (*Culiseta atra*, *Cx*. *australicus*, and *Cx*. *globocoxitus*). The closest reference genomes from the MalAvi database were the lineages COLL4, TERUF02 (*P. circumflexum*), BT7, and TURDUS1 (*P*. *homocircumflexum*) (GenBank: DQ368374, EU810618, MK062195, and AF495576, respectively). Lineages COLL4, TERUF02, and BT7 had a 21-nucleotide (4.4%) sequence divergence, whilst TURDUS1 had a 22-nucleotide (4.6%) sequence divergence from CULPER01 (Fig. 1; Supplementary Table S2).

The lineages CULPER02-03 were closely aligned to BELL01. CULPER02 was the most prevalent (*N* = 7), and diverged by one nucleotide from BELL01 (0.21%), with this SNP also being conserved in lineage CULPER03. CULPER03 diverged from BELL01 by 2 nucleotides (0.42%) and was only identified in an *Ae*. *notoscriptus* pool.

**Fig. 2.**
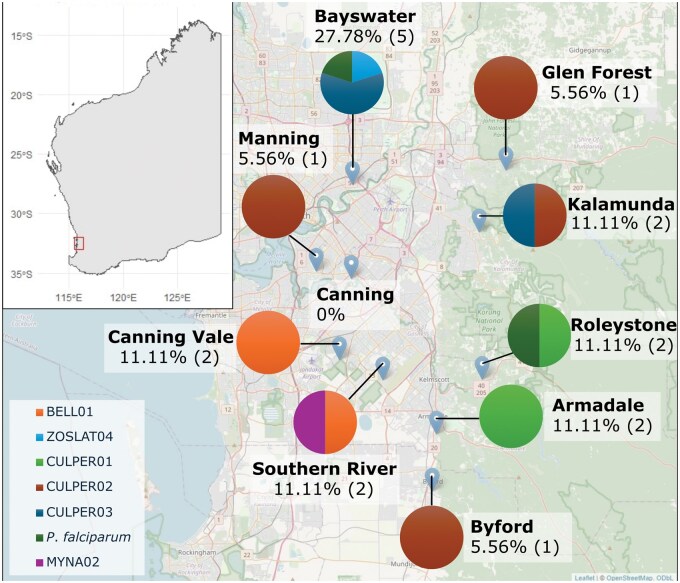
Location of the 10 sampling sites in Perth, Western Australia. The percentages and parenthesis numbers represent the prevalence of Haemosporida lineages at each sampling location. The proportion of each lineage at each location is visualized using pie charts. Line-age names are collected from the MalAvi database. Map was generated using Leaflet on RStudio.

Most trapping locations (90%, 9/10) were positive for at least one Haemosporida lineage over the study period (Fig. 2). BELL01 and closely related lineages (CULPER02-03) were widespread and identified at 7 locations, ranging from 1 to 3 detections per location. Bayswater had the highest number of parasite-positive mosquito pools and diversity, with 3 different *Plasmodium* spp. detected, including *P. falciparum*. CULPER01 was detected at 2 close locations (Armadale and Roleystone), located 7.75 km apart. BELL01 was detected at 2 locations (Canning Vale and Southern River), located 5.16 km apart. Detection of Haemosporida was consistent throughout the year; however, detection in *Aedes* spp. only occurred during the winter months (June to July) (Table 4).

**Table 4.** Total number of positive mosquito pools separated by sampling month

Month/year	Total positive (no. pools)	Genus	Positive per lineage (no.)	Lineage/species	Mosquito species
**Oct/2022**	5	*Plasmodium*	1	BELL01	*Cx*. *globocoxitus*
			2	CULPER01	*Culiseta atra*, *Cx*. *australicus*
			1	*falciparum*	*Cx*. *annulirostris*
		*Haemoproteus*	1	*zosteropis*	*Culiseta atra*
**Jan/2023**	0				
**Mar/2023**	0				
**April/2023**	4	*Plasmodium*	1	BELL01	*Cx*. *australicus*
			3	CULPER02	*Cx*. *australicus*, *Cx*. *globocoxitus*, *Cx*. *quinquefasciatus*
**June/2023**	1	*Plasmodium*	1	CULPER03	*Ae*. *notoscriptus*
**July/2023**	2	*Plasmodium*	1	CULPER01	*Cx*. *globocoxitus*
			1	CULPER02	*Ae*. *alboannulatus*
**Aug/2023**	0				
**Oct/2023**	2	*Plasmodium*	1	CULPER02	*Cx*. *australicus*
			1	MYNA02	*Cx*. *australicus*
**Jan/2024**	1	*Plasmodium*	1	*falciparum*	*Cx*. *annulirostris*
**Mar/2024**	0				
**April/2024**	0				
**June/2024**	0				
**July/2024**	2	*Plasmodium*	2	CULPER02	*Ae*. *alboannulatus*, *Cx*. *globocoxitus*
**Aug/2024**	1	*Plasmodium*	1	BELL01	*Cx*. *quinquefasciatus*

Results are reported by parasite genera, number of detections, lineage name, and subsequent mosquito pool species in which these were identified.

## Discussion

This is the first study to screen mosquitoes for blood-borne parasites in Western Australia, and to screen for avian Haemosporida in Australia. A diverse range of avian Haemosporida were identified from 3 mosquito genera across metropolitan Perth, Western Australia. Six avian Haemosporida lineages were identified, 3 of which had been previously reported, and 3 novel lineages (CULPER01-03). No filarial nematodes were detected.

The 3 previously identified lineages, one *Haemoproteus* (*H*. *zosteropis*) and 2 *Plasmodium* (BELL01 and MYNA02), have only been detected in the Oceania region, specifically New Zealand, New Caledonia, and eastern states of Australia (Baillie and Brunton 2011, Ewen et al. 2012, Zamora-Vilchis et al. 2012, Clark et al. 2015, Goulding et al. 2016, Schoener et al. 2020). Hence, these findings now extend their known geographical range to include Western Australia.

Each of the 3 previously identified lineages have only been detected in a limited range of avian species (Baillie and Brunton 2011, Ewen et al. 2012, Zamora-Vilchis et al. 2012, Clark et al. 2015, Goulding et al. 2016, Schoener et al. 2020), with this study expanding detections to include mosquitoes from the genera *Culex* for *Plasmodium*, and *Culiseta* for *Haemoproteus*. The local avian host species remains unknown, which limits the ability to infer the current risk to local host populations. A review of reported Haemosporida-infected avian populations revealed that, for each detected lineage, species from at least one Haemosporida-positive avian family reside within Perth. These estimates can be used to infer at-risk local avian hosts, although confirming the suspected avian-lineage association in Western Australia requires targeted screening of avian blood. Additionally, future studies using mosquito blood meal analysis (Gyawali et al. 2019, Guimarães et al. 2021) may also provide insight into mosquito ecology and identify at-risk host species.

The novel lineages CULPER01-03 demonstrate varying degrees of genetic divergence from known Haemosporida lineages. CULPER01 diverges by 4.4% to 4.6% from COLL4, TERUF02, BT7, and TURDUS. Hellgren et al. (2007) proposed that cyt *b* sequence divergence of 5% or more aligns with morphologically distinct species, with CULPER01 divergence falling just below this threshold. Interestingly, COLL4 and TURDUS1, which are considered morphologically distinct, differ by only 4.8%, which is also below the 5% threshold, suggesting CULPER01 may also represent a morphologically distinct lineage. This is plausible given the geographic isolation from related lineages, which have not been identified in the Oceanic region. However, morphological confirmation of this hypothesis requires identification and characterization in avian blood samples.

The cyt *b* gene region of avian Haemosporida is estimated to diverge by ∼1.2% per million years (Ricklefs and Outlaw 2010). Therefore, CULPER01’s 4.4% to 4.6% lineage divergence suggests divergence occurred approximately 3 to 4 million years ago, aligning with the Mid-Pliocene epoch (3 to 5 million years ago). During this period, the Nullarbor Plains, a now arid barrier that isolates the southwest of Australia, was a forested ecosystem with *Eucalyptus* and *Banksia* species. Forest landscapes would have allowed a migration corridor between the east and west of Australia. However, approximately 3 million years ago, this forest collapsed in response to Australia’s drying climate, giving rise to today’s Nullarbor Plain and isolating flora and fauna in the southwest (Sniderman et al. 2016). The estimated timing of CULPER01’s genetic divergence closely corresponds with this change in climate and landscape, suggesting it may represent a regionally endemic avian malaria species that evolved in isolation over millions of years in the southwest region of Australia.

In contrast, the novel lineages CULPER02-03 exhibit low sequence divergence (0.21% to 0.42%) from the known lineage BELL01, indicating recent evolutionary divergence. Detecting these closely related lineages is critical, as lineages with even one SNP may exhibit different host and geographical ranges (Beadell et al. 2004, Bensch et al. 2000, Reullier et al. 2006). CULPER02 was the most abundant and geographically widespread lineage, being detected at 5 sampling locations, primarily from species within the *Cx*. *pipiens* complex. Surprisingly, during the winter months, CULPER02-03 were identified within *Aedes* spp., which are not known to transmit avian *Plasmodium*. Detections in *Aedes* spp. potentially indicates a seasonal shift in mosquito feeding patterns to avian hosts for these generalist feeders (Kay et al. 2007, Webb et al. 2016).

The closest lineage to BELL01 on the MalAvi database, LICUN01, has also been detected in northern Western Australia. LICUN01 diverges from BELL01 by 1.5% (7 nucleotide difference) and was previously detected in the white-gaped honey eater (*Lichenostomus unicolor*, Meliphagidae) from the Kimberley region of Western Australia (Eastwood et al. 2019). BELL01 was similarly found in avian species within the Meliphagidae family, indicating that local species in this family may be the hosts for CULPER02-03 lineages (Baillie and Brunton 2011). Together with the discovery of CULPER02-03, these findings reveal a unique complex of BELL01-like lineages in Western Australia.

Not surprisingly, mosquito surveillance studies have identified a higher prevalence of avian Haemosporida in ornithophilic mosquito species (Ferraguti et al. 2013). Our study supports this finding as Haemosporida were primarily detected in mosquito species that preferentially feed from avian hosts (*Cx. australicus*, *C*x. *globocoxitus*, and *Cx. quinquefasciatus*) or are generalist feeders (*Cx. annulirostris*, *Ae. notoscriptus*, *Ae. alboannulatus*) (Johansen et al. 2009, Webb et al. 2016, Stephenson et al. 2019). Generalist feeders play a crucial role in amplifying and transmitting enzootic pathogens by facilitating the transmission of pathogens from infected hosts to other susceptible species, including humans (Weaver and Barrett 2004, Pierson and Diamond, 2020, Fikrig and Harrington 2021, Campos et al. 2023). Our detections of CULPER02-03 in *Ae*. spp. in winter months suggests that seasonal shifts in blood-feeding patterns can trigger enzootic transmission. The blood-feeding preference of *Culiseta atra* is currently understudied, with some evidence suggesting a preference for avian hosts (Johansen et al. 2009), highlighting the absence of literature on uncommon mosquito species. Further investigation is required to ascertain the potential role of *Culiseta atra* in the transmission of *Haemoproteus*.

Species within the *Cx*. *pipiens* complex are the suspected primary vectors of avian *Plasmodium* spp. (Schoener et al. 2017, Köchling et al. 2023, Garrigós et al. 2024). In this study, 75% of avian *Plasmodium* detections were from species within the *Cx. pipiens* complex (*Cx. australicus*, *Cx. globocoxitus*, *Cx. quinquefasciatus*), with the overall prevalence in *Culex pipiens* complex pools being 6.4%. Similar prevalence rates in *Cx*. *pipiens* complex populations have been observed worldwide, ranging from 2% to 20% (Reiter and LaPoite 2007, Kimura et al. 2010, Glaizot et al. 2012, Ferraguti et al. 2021, Garrigós et al. 2024). Vector competence for *Cx. pipiens* and *Cx. molestus* has been confirmed through avian *Plasmodium* detection in mosquito saliva post-blood feeding from infected birds (Gutiérrez-López et al. 2024). Although no *Cx. molestus* pools were positive for *Plasmodium* in this study, vector competence is thought to be lineage-dependent (Gutiérrez-López et al. 2024), suggesting *Cx. molestus* may not be competent for local parasite lineages. Alternatively, the blood feeding preference of *Cx. molestus* is inclined toward humans, which may explain this species’ lack of avian Haemosporida (Webb et al. 2016). However, it is important to note that species within the *Culex pipiens* complex (*Cx*. *australicus*, *Cx*. *globocoxitus*, *Cx*. *molestus*, and *Cx*. *quinquefasciatus*) exhibit ambiguity in both morphology and genetic barcoding, which can lead to potential misidentification (Batovska et al. 2016, Aardema et al. 2022).

Positive results from *Cx. annulirostris*, *Ae. alboannulatus*, *Ae. notoscriptus*, and *Culiseta atra* may be the result of mechanical transmission; however, given the lack of knowledge surrounding vector capacity, this cannot be confirmed, and the vector potential of each requires further study.

The detection of *P. falciparum* was unexpected but highlights the effectiveness of mosquito surveillance for public health purposes, as malaria is a notifiable infectious disease in Western Australia. Over the study period (2022 to 2024), the annual notification of human malaria infections in Perth ranged from 36 to 52 cases/years (Western Australian Notifiable Infectious Disease Dashboard, Accessed January 2025). Human malaria is consistently imported through travellers returning to Australia, with 58% of cases being positive for *P. falciparum* (Sohail et al. 2024). Perth, Western Australia, does not have competent vectors for *P*. *falciparum* transmission. Vectors for *P*. *falciparum* belong within the *Anopheles* genus; however, native *Anopheles* spp. (*An*. *annulipes* and *An*. *atripes*) are not competent vectors. *Culex* spp. prevent the full development of mammalian *Plasmodium* spp., therefore, this detection in *Cx*. *annulirostris* suggests mechanical transmission only and represents a dead-end host for *P. falciparum* (Knöckel et al. 2013, Molina-Cruz et al. 2013).

Numerous known vectors for the canine heartworm *D. immitis* (*Ae*. *notoscriptus*, *Ae*. *vigilax*, *Cx*. *annulirostris*, and *Cx*. *quinquefasciatus*) were collected in this study. However, *Dirofilaria* spp. were not detected with our molecular assays. This marks the first attempt to screen mosquitoes in Australia for *D*. *immitis* since 1985 (Russell 1985) and the only screening conducted in Western Australia. The true prevalence of *D*. *immitis* remains unknown in Western Australia, but it is predicted to be low (Atkinson et al. 2025). Therefore, although a negative result is not unexpected, these results cannot conclusively confirm the absence of *D*. *immitis* within the southwest of Australia.

## Conclusion

Molecular screening of mosquito populations for blood parasites has identified a significant diversity of avian Haemosporida within Perth, Western Australia. Six avian Haemosporida lineages were detected. Notably, 3 novel avian *Plasmodium* species were identified, with 3 closely aligned with the BELL01 lineage. The novel CULPER01 diverges significantly from its closest aligned lineages, suggesting that this lineage may have existed in the geographically isolated Oceania region for several million years and may constitute a new morphospecies. The predominance of avian *Plasmodium* prevalence in mosquitoes of the *Culex pipiens* complex reinforces their role as primary vectors in this region. The findings also suggest that local bird populations are primarily exposed to regionally endemic Haemosporida, likely shaped by Perth’s geographic isolation.

However, since this study only examined the south-eastern metropolitan areas of Perth, the diversity of Haemosporida found in mosquitoes is probably underestimated. Additionally, the pathogenicity of the identified lineages on Western Australia’s native bird populations remains unknown. Therefore, expanding surveillance efforts to include both mosquito and bird populations is advised to better understand the full range of Haemosporida diversity and the potential impact on native host species.

## Supplementary Material

tjaf142_Supplementary_Data
