# Supplementary material for: Molecular detection of avian parasites in Australian mosquitoes (Culicidae)
Source: J Med Entomol. 2025 Oct 7;62(6):1599–610. doi: 10.1093/jme/tjaf142 (PMC12616232; doi:10.1093/jme/tjaf142)
Supplement: tjaf142_Supplementary_Data [file tjaf142_supplementary_data.zip › Peck_SuppTable2.docx]

**Supplementary Table 2:** Regional and host family information of the avian *Plasmodium* lineages with the closest alignment to novel CULPER01 detected in Perth, Western Australia.

| Lineage  (Accession no.) | The host family previously identified | Previous regions identified | Reference |
| --- | --- | --- | --- |
| BT7  (AY393793) | **Accipitridae**, **Anatidae**, Certhiidae, **Charadriidae**, **Corvidae**, Fringillidae, **Hirundinidae**, Mimidae, **Motacillidae**, Muscicapidae, Paridae, Parulidae, **Phasianidae**, **Scolopacidae**, Sylviidae, Turdidae | Asia, Europe, North America, South America | Beadell et al. 2006, Cosgrove et al. 2008, Cozzarolo et al. 2018, DeBrock et al. 2021, Dodge et al. 2013, Dubiec et al. 2016, Ellis et al. 2020, Fecchio et al. 2023, Galen et al. 2018, Harl et al. 2022, Hellgren 2005, Hellgren et al. 2007, Henschen et al. 2017, Huang et al. 2015, Huang et al. 2020, Inumaru et al. 2022, Ishtiaq et al. 2007, Jones et al. 2018, Kim et al. 2009, Knowles et al. 2011, Krone et al. 2008, Loiseau et al. 2012, Lynton-Jenkins et al. 2020, Martínez-Renau et al. 2022, Martinsen et al. 2008, Neto et al. 2020, Oakgrove et al. 2014, Outlaw and Ricklefs 2009, Pagenknopp et al. 2008, Palinauskas et al. 2013, Podmokla et al. 2014a, Podmokla et al. 2014b, Pulgarin-R et al. 2019, Ramey et al. 2012, Ramey et al. 2013, Ramey et al. 2016, Ramey et al. 2021, Smith et al. 2016, Smith et al. 2018, Stjernman et al. 2008, Šujanová et al. 2021, Svoboda et al. 2015, Szöllosi et al. 2011, Turcotte et al. 2018, Van Hemert et al. 2019, van Rooyen et al. 2013, Walther et al. 2016, Wilkinson et al. 2016, Wood et al. 2007, Yohannes et al. 2009, Yusupova et al. 2023 |
| Lineage  (Accession no.) | **The host family previously identified** | **Previous regions identified** | **Reference** |
| TURDUS1  (AF495576) | **Accipitridae**, Certhiidae, **Corvidae**, Fringillidae, **Hirundinidae**, **Motacillidae**, Muscicapidae, Paridae, **Passeridae**, **Scolopacidae**, Sittidae, **Strigidae**, Sylviidae, Turdidae | Africa, Asia, Europe | Cosgrove et a.l 2008, Dimitrov et al. 2010, Dimitrov et al. 2010, Dubiec et al. 2016, Ellis et al. 2020, Fletcher et al. 2019, Glaizot et al. 2012, Hanel et al. 2016, Harl et al. 2020, Harl et al. 2022, Hellgren 2005, Hellgren et al. 2007, Hellgren et al. 2013, Huang et al. 2015, Huang et al. 2020, Isaksson et al. 2013, Jones et al. 2018, Jones et al. 2024, Knowles et al. 2011, Krizanauskiene et al. 2006, Krone et al. 2008, Kubacka et al. 2019, Kulma et al. 2013, Kulma et al. 2014, Loiseau et al. 2011, Lynton-Jenkins et al. 2020, Mendes et al. 2013, Neto et al. 2020, Nilsson et al. 2016, Palinauskas et al. 2007, Palinauskas et al. 2013, Palinauskas et al. 2015, Pérez-Rodriguez et al. 2015, Piersma and van der Velde 2012, Podmokla et al. 2014a, Podmokla et al. 2014b, Santiago-Alarcon et al. 2016, Schumm et al. 2019, Schumm et al. 2019, Stjernman et al. 2008, Strehmann et al. 2023, Sujanová et al. 2021, Svoboda et al. 2015, Synek et al. 2013, Synek et al. 2016, Szöllosi et al. 2011, Tanigawa et al. 2013, Valkiunas et al. 2022, van Rooyen et al. 2013, von Rönn et al. 2015, Waldenström et al. 2002, Wiersch et al. 2007, Wiersch et al. 2007, Wood et al. 2007, Yusupova et al. 2023 |
| Lineage  (Accession no.) | **The host family previously identified** | **Previous regions identified** | **Reference** |
| COLL4  (DQ368374) | Fringillidae, Furnariidae, Icteridae, Laniidae, Mimidae, Muscicapidae, Ploceidae, Pycnonotidae, **Sturnidae**, Turdidae, Tyrannidae, Vireonidae | Africa, Asia, Europe, North America, South America | DeBrock et al. 2021, Doussang et al. 2021, Durrant et al. 2006, Fecchio et al. 2019, Fecchio et al. 2021, Garamszegi et al. 2015, Harvey and Voelker 2017, Ilgunas et al. 2016, Jones et al. 2018, Kulma et al. 2013, Kulma et al. 2014, Levin et al. 2013, Musa et al. 2019, Musa et al. 2022, Palinauskas et al. 2015, Rodrigues et al. 2021, Szöllösi et al. 2016 |
| TERUF02  (EU810618) | **Monarchidae**, Muscicapidae **Scolopacidae** | Africa, Asia, Europe | Beadell et al. 2009, Garamszegi et al. 2015, Jones et al. 2018, Kulma et al. 2013, Kulma et al. 2014, Mendes et al. 2013, Szöllösi et al. 2016 |

Bolded families indicate that species within that family reside in Perth, Western Australia.

## References:

Beadell JS, Covas R, Gebhard C, Ishtiaq F, Melo M, Schmidt BK, Perkins SL, Graves GR, Fleischer RC. 2009. Host associations and evolutionary relationships of avian blood parasites from West Africa. Int J Parasitol. 39(2):257–266. doi:[10.1016/j.ijpara.2008.06.005](https://doi.org/10.1016/j.ijpara.2008.06.005).

Beadell JS, Ishtiaq F, Covas R, Melo M, Warren BH, Atkinson CT, Bensch S, Graves GR, Jhala YV, Peirce MA, et al. 2006. Global phylogeographic limits of Hawaii’s avian malaria. Proceedings of the  R Soc Biol Sci. 273(1604):2935–2944. doi:[10.1098/rspb.2006.3671](https://doi.org/10.1098/rspb.2006.3671).

Cosgrove CL, Wood MJ, Day KP, Sheldon BC. 2008. Seasonal variation in Plasmodium prevalence in a population of blue tits Cyanistes caeruleus. J Anim Ecol. 77(3):540–548. doi:[10.1111/j.1365-2656.2008.01370.x](https://doi.org/10.1111/j.1365-2656.2008.01370.x).

Cozzarolo C-S, Jenkins T, Toews DPL, Brelsford A, Christe P. 2018. Prevalence and diversity of haemosporidian parasites in the yellow-rumped warbler hybrid zone. Ecol Evol. 8(19):9834–9847. doi:[10.1002/ece3.4469](https://doi.org/10.1002/ece3.4469).

DeBrock S, Cohen E, Balasubramanian S, Marra PP, Hamer SA. 2021. Characterization of the Plasmodium and Haemoproteus parasite community in temperate-tropical birds during spring migration. Int J Parasitol Parasites Wildl. 15:12. doi:[10.1016/j.ijppaw.2021.03.013](https://doi.org/10.1016/j.ijppaw.2021.03.013).

Dimitrov D, Zehtindjiev P, Bensch S. 2010. Genetic diversity of avian blood parasites in SE Europe: Cytochrome b lineages of the genera Plasmodium and Haemoproteus (Haemosporida) from Bulgaria. Acta Parasit. 55(3):201–209. doi:[10.2478/s11686-010-0029-z](https://doi.org/10.2478/s11686-010-0029-z).

Dodge M, Guers SL, Sekercioğlu ÇH, Sehgal RNM. 2013. North American Transmission of Hemosporidian Parasites in the Swainson’s Thrush (Catharus ustulatus), a Migratory Songbird. J Parasitol. 99(3):548–553.

Doussang D, Sallaberry-Pincheira N, Cabanne GS, Lijtmaer DA, González-Acuña D, Vianna JA. 2021. Specialist versus generalist parasites: the interactions between host diversity, environment and geographic barriers in avian malaria. Int J Parasitol. 51(11):899–911. doi:[10.1016/j.ijpara.2021.04.003](https://doi.org/10.1016/j.ijpara.2021.04.003).

Dubiec A, Podmokła E, Zagalska-Neubauer M, Drobniak SM, Arct A, Gustafsson L, Cichoń M. 2016. Differential prevalence and diversity of haemosporidian parasites in two sympatric closely related non-migratory passerines. Parasitology. 143(10):1320–1329. doi:[10.1017/S0031182016000779](https://doi.org/10.1017/S0031182016000779).

Durrant KL, Beadell JS, Ishtiaq F, Graves GR, Olson SL, Gering E, Peirce MA, Milensky CM, Schmidt BK, Gebhard C, et al. 2006. Avian Hematozoa in South America: a Comparison of Temperate and Tropical Zones. In: Ornithological Monographs No. 60. Vol. No. 60. American Ornithological Society. p. 98. [accessed 2025 Feb 11]. <https://bioone.org/ebooks/Ornithological-Monographs/Ornithological-Monographs-No-60/9/Avian-Hematozoa-in-South-America--a-Comparison-of-Temperate/10.2307/40166831>.

Ellis VA, Huang X, Westerdahl H, Jönsson J, Hasselquist D, Neto JM, Nilsson J-Å, Nilsson J, Hegemann A, Hellgren O, et al. 2020. Explaining prevalence, diversity and host specificity in a community of avian haemosporidian parasites. Oikos. 129(9):1314–1329. doi:[10.1111/oik.07280](https://doi.org/10.1111/oik.07280).

Fecchio A, Bell JA, Pinheiro RBP, Cueto VR, Gorosito CA, Lutz HL, Gaiotti MG, Paiva LV, França LF, Toledo-Lima G, et al. 2019. Avian host composition, local speciation and dispersal drive the regional assembly of avian malaria parasites in South American birds. Mol Ecol. 28(10):2681–2693. doi:[10.1111/mec.15094](https://doi.org/10.1111/mec.15094).

Fecchio A, Bell JA, Williams EJ, Dispoto JH, Weckstein JD, de Angeli Dutra D. 2023. Co-infection with Leucocytozoon and Other Haemosporidian Parasites Increases with Latitude and Altitude in New World Bird Communities. Microb Ecol. 86(4):2838–2846. doi:[10.1007/s00248-023-02283-x](https://doi.org/10.1007/s00248-023-02283-x).

Fecchio A, Lima MR, Bell JA, Schunck F, Corrêa AH, Beco R, Jahn AE, Fontana CS, da Silva TW, Repenning M, et al. 2021. Loss of forest cover and host functional diversity increases prevalence of avian malaria parasites in the Atlantic Forest. Int J Parasitol . 51(9):719–728. doi:[10.1016/j.ijpara.2021.01.001](https://doi.org/10.1016/j.ijpara.2021.01.001).

Fletcher K, Träff J, Gustafsson L. 2019. Importance of infection of haemosporidia blood parasites during different life history stages for long-term reproductive fitness of collared flycatchers. J Avian Biol. 50(8). doi:[10.1111/jav.02118](https://doi.org/10.1111/jav.02118). [accessed 2025 Feb 11]. <https://onlinelibrary.wiley.com/doi/abs/10.1111/jav.02118>.

Galen SC, Nunes R, Sweet PR, Perkins SL. 2018. Integrating coalescent species delimitation with analysis of host specificity reveals extensive cryptic diversity despite minimal mitochondrial divergence in the malaria parasite genus Leucocytozoon. BMC Evol Biol. 18(1):128. doi:[10.1186/s12862-018-1242-x](https://doi.org/10.1186/s12862-018-1242-x).

Garamszegi LZ, Zagalska-Neubauer M, Canal D, Markó G, Szász E, Zsebők S, Szöllősi E, Herczeg G, Török J. 2015. Malaria parasites, immune challenge, MHC variability, and predator avoidance in a passerine bird. Behav Ecol. 26(5):1292–1302. doi:[10.1093/beheco/arv077](https://doi.org/10.1093/beheco/arv077).

Harl J, Himmel T, Valkiūnas G, Ilgūnas M, Bakonyi T, Weissenböck H. 2020. Geographic and host distribution of haemosporidian parasite lineages from birds of the family Turdidae. Malar J. 19(1):335. doi:[10.1186/s12936-020-03408-0](https://doi.org/10.1186/s12936-020-03408-0).

Harl J, Himmel T, Valkiūnas G, Ilgūnas M, Nedorost N, Matt J, Kübber-Heiss A, Alic A, Konicek C, Weissenböck H. 2022. Avian haemosporidian parasites of accipitriform raptors. Malar J. 21(1):14. doi:[10.1186/s12936-021-04019-z](https://doi.org/10.1186/s12936-021-04019-z).

Harvey JA, Voelker G. 2017. Avian haemosporidian detection across source materials: prevalence and genetic diversity. Parasitol Res. 116(12):3361–3371. doi:[10.1007/s00436-017-5654-0](https://doi.org/10.1007/s00436-017-5654-0).

Hellgren O. 2005. The occurrence of haemosporidian parasites in the Fennoscandian bluethroat (Luscinia svecica) population. J Ornithol. 146(1):55–60. doi:[10.1007/s10336-004-0055-4](https://doi.org/10.1007/s10336-004-0055-4).

Hellgren O, Kutzer M, Bensch S, Valkiūnas G, Palinauskas V. 2013. Identification and characterization of the merozoite surface protein 1 (msp1) gene in a host-generalist avian malaria parasite, Plasmodium relictum (lineages SGS1 and GRW4) with the use of blood transcriptome. Malar J. 12:381. doi:[10.1186/1475-2875-12-381](https://doi.org/10.1186/1475-2875-12-381).

Hellgren O, Waldenström J, Peréz-Tris J, Szöll E, Si Ö, Hasselquist D, Krizanauskiene A, Ottosson U, Bensch S. 2007. Detecting shifts of transmission areas in avian blood parasites — a phylogenetic approach. Mol Ecol. 16(6):1281–1290. doi:[10.1111/j.1365-294X.2007.03227.x](https://doi.org/10.1111/j.1365-294X.2007.03227.x).

Henschen AE, Whittingham LA, Dunn PO. 2017. The relationship between blood parasites and ornamentation depends on the level of analysis in the common yellowthroat. J Avian Biol. 48(9):1263–1272. doi:[10.1111/jav.01418](https://doi.org/10.1111/jav.01418).

Huang X, Dong L, Zhang C, Zhang Y. 2015. Genetic diversity, temporal dynamics, and host specificity in blood parasites of passerines in north China. Parasitol Res. 114(12):4513–4520. doi:[10.1007/s00436-015-4695-5](https://doi.org/10.1007/s00436-015-4695-5).

Huang X, Huang D, Liang Y, Zhang L, Yang G, Liu B, Peng Y, Deng W, Dong L. 2020. A new protocol for absolute quantification of haemosporidian parasites in raptors and comparison with current assays. Parasit Vectors. 13(1):354. doi:[10.1186/s13071-020-04195-y](https://doi.org/10.1186/s13071-020-04195-y).

Ilgūnas M, Bukauskaitė D, Palinauskas V, Iezhova TA, Dinhopl N, Nedorost N, Weissenbacher-Lang C, Weissenböck H, Valkiūnas G. 2016. Mortality and pathology in birds due to Plasmodium (Giovannolaia) homocircumflexum infection, with emphasis on the exoerythrocytic development of avian malaria parasites. Malar J. 15(1):256. doi:[10.1186/s12936-016-1310-x](https://doi.org/10.1186/s12936-016-1310-x).

Inumaru M, Nishiumi I, Kawakami K, Sato Y. 2022. A widespread survey of avian haemosporidia in deceased wild birds of Japan: the hidden value of personally collected samples. J Vet Med Sci. 84(9):1253–1260. doi:[10.1292/jvms.22-0179](https://doi.org/10.1292/jvms.22-0179).

Ishtiaq F, Gering E, Rappole JH, Rahmani AR, Jhala YV, Dove CJ, Milensky C, Olson SL, Peirce MA, Fleischer RC. 2007. Prevalence and diversity of avian hematozoan parasites in Asia: a regional survey. J Wildl Dis. 43(3):382–398. doi:[10.7589/0090-3558-43.3.382](https://doi.org/10.7589/0090-3558-43.3.382).

Jones W, Kulma K, Bensch S, Cichoń M, Kerimov A, Krist M, Laaksonen T, Moreno J, Munclinger P, Slater FM, et al. 2018. Interspecific transfer of parasites following a range-shift in Ficedula flycatchers. Ecol Evol. 8(23):12183–12192. doi:[10.1002/ece3.4677](https://doi.org/10.1002/ece3.4677).

Jones W, Reifová R, Reif J, Synek P, Šíma M, Munclinger P. 2024. Sympatry in a nightingale contact zone has no effect on host-specific blood parasite prevalence and lineage diversity. Int J Parasitol. 54(7):357–366. doi:[10.1016/j.ijpara.2024.03.002](https://doi.org/10.1016/j.ijpara.2024.03.002).

Kim KS, Tsuda Y, Sasaki T, Kobayashi M, Hirota Y. 2009. Mosquito blood-meal analysis for avian malaria study in wild bird communities: laboratory verification and application to Culex sasai (Diptera: Culicidae) collected in Tokyo, Japan. Parasitol Res. 105(5):1351–1357. doi:[10.1007/s00436-009-1568-9](https://doi.org/10.1007/s00436-009-1568-9).

Knowles SCL, Wood MJ, Alves R, Wilkin TA, Bensch S, Sheldon BC. 2011. Molecular epidemiology of malaria prevalence and parasitaemia in a wild bird population. Mol Ecol. 20(5):1062–1076. doi:[10.1111/j.1365-294X.2010.04909.x](https://doi.org/10.1111/j.1365-294X.2010.04909.x).

Krizanauskiene A, Hellgren O, Kosarev V, Sokolov L, Bensch S, Valkiunas G. 2006. Variation in host specificity between species of avian hemosporidian parasites: evidence from parasite morphology and cytochrome B gene sequences. J Parasitol. 92(6):1319–1324. doi:[10.1645/GE-873R.1](https://doi.org/10.1645/GE-873R.1).

Krone O, Waldenström J, Valkiūnas G, Lessow O, Müller K, Lezhova TA, Fickel J, Bensch S. 2008. Haemosporidian Blood Parasites in European Birds of Prey and Owls. J Parasitol. 94(3):709–715.

Kubacka J, Gerlée A, Foucher J, Korb J, Podmokła E. 2019. Correlates of blood parasitism in a threatened marshland passerine: infection by kinetoplastids of the genus Trypanosoma is related to landscape metrics of habitat edge. Parasitology. 146(8):1036–1046. doi:[10.1017/S0031182019000350](https://doi.org/10.1017/S0031182019000350).

Kulma K, Low M, Bensch S, Qvarnström A. 2013. Malaria infections reinforce competitive asymmetry between two Ficedula flycatchers in a recent contact zone. Mol Ecol. 22(17):4591–4601. doi:[10.1111/mec.12409](https://doi.org/10.1111/mec.12409).

Kulma K, Low M, Bensch S, Qvarnström A. 2014. Malaria-Infected Female Collared Flycatchers (Ficedula albicollis) Do Not Pay the Cost of Late Breeding. PLOS ONE. 9(1):e85822. doi:[10.1371/journal.pone.0085822](https://doi.org/10.1371/journal.pone.0085822).

Levin II, Zwiers P, Deem SL, Geest EA, Higashiguchi JM, Iezhova TA, Jiménez-Uzcátegui G, Kim DH, Morton JP, Perlut NG, et al. 2013. Multiple lineages of Avian malaria parasites (Plasmodium) in the Galapagos Islands and evidence for arrival via migratory birds. Conserv Biol. 27(6):1366–1377. doi:[10.1111/cobi.12127](https://doi.org/10.1111/cobi.12127).

Loiseau C, Harrigan RJ, Cornel AJ, Guers SL, Dodge M, Marzec T, Carlson JS, Seppi B, Sehgal RNM. 2012. First Evidence and Predictions of Plasmodium Transmission in Alaskan Bird Populations. PLOS ONE. 7(9):e44729. doi:[10.1371/journal.pone.0044729](https://doi.org/10.1371/journal.pone.0044729).

Loiseau C, Zoorob R, Robert A, Chastel O, Julliard R, Sorci G. 2011. Plasmodium relictum infection and MHC diversity in the house sparrow (Passer domesticus). Proc Biol Sci. 278(1709):1264–1272. doi:[10.1098/rspb.2010.1968](https://doi.org/10.1098/rspb.2010.1968).

Lynton-Jenkins JG, Bründl AC, Cauchoix M, Lejeune LA, Sallé L, Thiney AC, Russell AF, Chaine AS, Bonneaud C. 2020. Contrasting the seasonal and elevational prevalence of generalist avian haemosporidia in co-occurring host species. Ecol Evol. 10(12):6097–6111. doi:[10.1002/ece3.6355](https://doi.org/10.1002/ece3.6355).

Martínez-Renau E, Rojas-Estévez N, Friis G, Hernández-Montoya JC, Elizondo P, Milá B. 2022. Haemosporidian parasite diversity and prevalence in the songbird genus Junco across Central and North America. Ornithology. 139(3):ukac022. doi:[10.1093/ornithology/ukac022](https://doi.org/10.1093/ornithology/ukac022).

Martinsen ES, Perkins SL, Schall JJ. 2008. A three-genome phylogeny of malaria parasites (*Plasmodium* and closely related genera): Evolution of life-history traits and host switches. Mol Phylogenet Evol. 47(1):261–273. doi:[10.1016/j.ympev.2007.11.012](https://doi.org/10.1016/j.ympev.2007.11.012).

Mata VA, da Silva LP, Lopes RJ, Drovetski SV. 2015. The Strait of Gibraltar poses an effective barrier to host-specialised but not to host-generalised lineages of avian Haemosporidia. Int J Parasitol. 45(11):711–719. doi:[10.1016/j.ijpara.2015.04.006](https://doi.org/10.1016/j.ijpara.2015.04.006).

McNew SM, Barrow LN, Williamson JL, Galen SC, Skeen HR, DuBay SG, Gaffney AM, Johnson AB, Bautista E, Ordoñez P, et al. 2021. Contrasting drivers of diversity in hosts and parasites across the tropical Andes. Proc Natl Acad Sci U S A. 118(12):e2010714118. doi:[10.1073/pnas.2010714118](https://doi.org/10.1073/pnas.2010714118).

Mendes L, Pardal S, Morais J, Antunes S, Ramos JA, Perez-Tris J, Piersma T. 2013a. Hidden haemosporidian infections in Ruffs (Philomachus pugnax) staging in Northwest Europe en route from Africa to Arctic Europe. Parasitol Res. 112(5):2037–2043. doi:[10.1007/s00436-013-3362-y](https://doi.org/10.1007/s00436-013-3362-y).

Mendes L, Pardal S, Morais J, Antunes S, Ramos JA, Perez-Tris J, Piersma T. 2013b. Hidden haemosporidian infections in Ruffs (Philomachus pugnax) staging in Northwest Europe en route from Africa to Arctic Europe. Parasitol Res. 112(5):2037–2043. doi:[10.1007/s00436-013-3362-y](https://doi.org/10.1007/s00436-013-3362-y).

Musa S, Mackenstedt U, Woog F, Dinkel A. 2019. Avian malaria on Madagascar: prevalence, biodiversity and specialization of haemosporidian parasites. Int J Parasitol. 49(3–4):199–210. doi:[10.1016/j.ijpara.2018.11.001](https://doi.org/10.1016/j.ijpara.2018.11.001).

Musa S, Mackenstedt U, Woog F, Dinkel A. 2022. Untangling the actual infection status: detection of avian haemosporidian parasites of three Malagasy bird species using microscopy, multiplex PCR, and nested PCR methods. Parasitol Res. 121(10):2817–2829. doi:[10.1007/s00436-022-07606-4](https://doi.org/10.1007/s00436-022-07606-4).

Neto JM, Mellinger S, Halupka L, Marzal A, Zehtindjiev P, Westerdahl H. 2020. Seasonal dynamics of haemosporidian (Apicomplexa, Haemosporida) parasites in house sparrows *Passer domesticus* at four European sites: comparison between lineages and the importance of screening methods. Int J Parasitol. 50(6):523–532. doi:[10.1016/j.ijpara.2020.03.008](https://doi.org/10.1016/j.ijpara.2020.03.008).

Nilsson E, Taubert H, Hellgren O, Huang X, Palinauskas V, Markovets MY, Valkiūnas G, Bensch S. 2016. Multiple cryptic species of sympatric generalists within the avian blood parasite Haemoproteus majoris. J Evol Biol. 29(9):1812–1826. doi:[10.1111/jeb.12911](https://doi.org/10.1111/jeb.12911).

Oakgrove KS, Harrigan RJ, Loiseau C, Guers S, Seppi B, Sehgal RNM. 2014. Distribution, diversity and drivers of blood-borne parasite co-infections in Alaskan bird populations. Int J Parasitol. 44(10):717–727. doi:[10.1016/j.ijpara.2014.04.011](https://doi.org/10.1016/j.ijpara.2014.04.011).

Olias P, Wegelin M, Zenker W, Freter S, Gruber AD, Klopfleisch R. 2011. Avian Malaria Deaths in Parrots, Europe. Emerg Infect Dis. 17(5):950–952. doi:[10.3201/eid1705.101618](https://doi.org/10.3201/eid1705.101618).

Outlaw DC, Ricklefs RE. 2009. On the phylogenetic relationships of haemosporidian parasites from raptorial birds (Falconiformes and Strigiformes). J Parasitol. 95(5):1171–1176. doi:[10.1645/GE-1982.1](https://doi.org/10.1645/GE-1982.1).

Pagenkopp KM, Klicka J, Durrant KL, Garvin JC, Fleischer RC. 2008. Geographic variation in malarial parasite lineages in the common yellowthroat (Geothlypis trichas). Conserv Genet. 9(6):1577–1588. doi:[10.1007/s10592-007-9497-6](https://doi.org/10.1007/s10592-007-9497-6).

Palinauskas V, Iezhova TA, Križanauskienė A, Markovets MYu, Bensch S, Valkiūnas G. 2013. Molecular characterization and distribution of *Haemoproteus minutus* (Haemosporida, Haemoproteidae): A pathogenic avian parasite. Parasitol Int. 62(4):358–363. doi:[10.1016/j.parint.2013.03.006](https://doi.org/10.1016/j.parint.2013.03.006).

Palinauskas V, Žiegytė R, Ilgūnas M, Iezhova TA, Bernotienė R, Bolshakov C, Valkiūnas G. 2015. Description of the first cryptic avian malaria parasite, *Plasmodium homocircumflexum* n. sp., with experimental data on its virulence and development in avian hosts and mosquitoes. Int J Parasitol. 45(1):51–62. doi:[10.1016/j.ijpara.2014.08.012](https://doi.org/10.1016/j.ijpara.2014.08.012).

Pérez-Rodríguez A, de la Hera I, Bensch S, Pérez-Tris J. 2015. Evolution of seasonal transmission patterns in avian blood-borne parasites. Int J Parasitol. 45(9):605–611. doi:[10.1016/j.ijpara.2015.03.008](https://doi.org/10.1016/j.ijpara.2015.03.008).

Piersma T, van der Velde M. 2012. Dutch House Martins Delichon urbicum gain blood parasite infections over their lifetime, but do not seem to suffer. J Ornithol. 153(3):907–912. doi:[10.1007/s10336-012-0826-2](https://doi.org/10.1007/s10336-012-0826-2).

Podmokła E, Dubiec A, Drobniak SM, Arct A, Gustafsson L, Cichoń M. 2014a. Avian malaria is associated with increased reproductive investment in the blue tit. J Avian Biol. 45(3):219–224. doi:[10.1111/j.1600-048X.2013.00284.x](https://doi.org/10.1111/j.1600-048X.2013.00284.x).

Podmokła E, Dubiec A, Drobniak SM, Arct A, Gustafsson L, Cichoń M. 2014b. Determinants of prevalence and intensity of infection with malaria parasites in the Blue Tit. J Ornithol. 155(3):721–727. doi:[10.1007/s10336-014-1058-4](https://doi.org/10.1007/s10336-014-1058-4).

Pulgarín-R PC, Gómez C, Bayly NJ, Bensch S, FitzGerald AM, Starkloff N, Kirchman JJ, González-Prieto AM, Hobson KA, Ungvari-Martin J, et al. 2019. Migratory birds as vehicles for parasite dispersal? Infection by avian haemosporidians over the year and throughout the range of a long-distance migrant. J Biogeogr. 46(1):83–96. doi:[10.1111/jbi.13453](https://doi.org/10.1111/jbi.13453).

Ramey AM, Buchheit RM, Uher-Koch BD, Reed JA, Pacheco MA, Escalante AA, Schmutz JA. 2021. Negligible evidence for detrimental effects of *Leucocytozoon* infections among Emperor Geese (*Anser canagicus*) breeding on the Yukon-Kuskokwim Delta, Alaska. Int J Parasitol Parasites Wildl. 16:103–112. doi:[10.1016/j.ijppaw.2021.08.006](https://doi.org/10.1016/j.ijppaw.2021.08.006).

Ramey AM, Ely CR, Schmutz JA, Pearce JM, Heard DJ. 2012. Molecular Detection of Hematozoa Infections in Tundra Swans Relative to Migration Patterns and Ecological Conditions at Breeding Grounds. PLOS ONE. 7(9):e45789. doi:[10.1371/journal.pone.0045789](https://doi.org/10.1371/journal.pone.0045789).

Ramey AM, Fleskes JP, Schmutz JA, Yabsley MJ. 2013. Evaluation of blood and muscle tissues for molecular detection and characterization of hematozoa infections in northern pintails (*Anas acuta*) wintering in California. Int J Parasitol Parasites Wildl2:102–109. doi:[10.1016/j.ijppaw.2013.02.001](https://doi.org/10.1016/j.ijppaw.2013.02.001).

Ramey AM, Reed JA, Walther P, Link P, Schmutz JA, Douglas DC, Stallknecht DE, Soos C. 2016. Evidence for the exchange of blood parasites between North America and the Neotropics in blue-winged teal (Anas discors). Parasitol Res. 115(10):3923–3939. doi:[10.1007/s00436-016-5159-2](https://doi.org/10.1007/s00436-016-5159-2).

Santiago-Alarcon D, MacGregor-Fors I, Kühnert K, Segelbacher G, Schaefer HM. 2016. Avian haemosporidian parasites in an urban forest and their relationship to bird size and abundance. Urban Ecosyst. 19(1):331–346. doi:[10.1007/s11252-015-0494-0](https://doi.org/10.1007/s11252-015-0494-0).

Schumm YR, Wecker C, Marek C, Wassmuth M, Bentele A, Willems H, Reiner G, Quillfeldt P. 2019. Blood parasites in Passeriformes in central Germany: prevalence and lineage diversity of Haemosporida (Haemoproteus, Plasmodium and Leucocytozoon) in six common songbirds. PeerJ. 6:e6259. doi:[10.7717/peerj.6259](https://doi.org/10.7717/peerj.6259).

Smith JD, Gill SA, Baker KM, Vonhof MJ. 2018. Prevalence and diversity of avian Haemosporida infecting songbirds in southwest Michigan. Parasitol Res. 117(2):471–489. doi:[10.1007/s00436-017-5724-3](https://doi.org/10.1007/s00436-017-5724-3).

Smith MM, Van Hemert C, Merizon R. 2016. Haemosporidian parasite infections in grouse and ptarmigan: Prevalence and genetic diversity of blood parasites in resident Alaskan birds. Int J Parasitol Parasites Wildl. 5(3):229–239. doi:[10.1016/j.ijppaw.2016.07.003](https://doi.org/10.1016/j.ijppaw.2016.07.003).

Stjernman M, Råberg L, Nilsson J-Å. 2008. Maximum Host Survival at Intermediate Parasite Infection Intensities. PLOS ONE. 3(6):e2463. doi:[10.1371/journal.pone.0002463](https://doi.org/10.1371/journal.pone.0002463).

Strehmann F, Becker M, Lindner K, Masello JF, Quillfeldt P, Schumm YR, Farwig N, Schabo DG, Rösner S. 2023. Half of a forest bird community infected with haemosporidian parasites. Front Ecol Evol. 11. doi:[10.3389/fevo.2023.1107736](https://doi.org/10.3389/fevo.2023.1107736). [accessed 2025 Feb 11]. <https://www.frontiersin.org/journals/ecology-and-evolution/articles/10.3389/fevo.2023.1107736/full>.

Šujanová A, Špitalská E, Václav R. 2021. Seasonal Dynamics and Diversity of Haemosporidians in a Natural Woodland Bird Community in Slovakia. Diversity. 13(9):439. doi:[10.3390/d13090439](https://doi.org/10.3390/d13090439).

Svoboda A, Marthinsen G, Pavel V, Chutný B, Turčoková L, Lifjeld JT, Johnsen A. 2015. Blood parasite prevalence in the Bluethroat is associated with subspecies and breeding habitat. J Ornithol. 156(2):371–380. doi:[10.1007/s10336-014-1134-9](https://doi.org/10.1007/s10336-014-1134-9).

Synek P, Albrecht T, Vinkler M, Schnitzer J, Votýpka J, Munclinger P. 2013. Haemosporidian parasites of a European passerine wintering in South Asia: diversity, mixed infections and effect on host condition. Parasitol Res. 112(4):1667–1677. doi:[10.1007/s00436-013-3323-5](https://doi.org/10.1007/s00436-013-3323-5).

Synek P, Popelková A, Koubínová D, Šťastný K, Langrová I, Votýpka J, Munclinger P. 2016. Haemosporidian infections in the Tengmalm’s Owl (Aegolius funereus) and potential insect vectors of their transmission. Parasitol Res. 115(1):291–298. doi:[10.1007/s00436-015-4745-z](https://doi.org/10.1007/s00436-015-4745-z).

Szöllősi E, Cichoń M, Eens M, Hasselquist D, Kempenaers B, Merino S, Nilsson J ‐Å., Rosivall B, Rytkönen S, TÖRÖK J, et al. 2011. Determinants of distribution and prevalence of avian malaria in blue tit populations across Europe: separating host and parasite effects. J Evol Biol. 24(9):2014–2024. doi:[10.1111/j.1420-9101.2011.02339.x](https://doi.org/10.1111/j.1420-9101.2011.02339.x).

Szöllősi E, Garamszegi LZ, Hegyi G, Laczi M, Rosivall B, Török J. 2016. Haemoproteus infection status of collared flycatcher males changes within a breeding season. Parasitol Res. 115(12):4663–4672. doi:[10.1007/s00436-016-5258-0](https://doi.org/10.1007/s00436-016-5258-0).

Tanigawa M, Sato Y, Ejiri H, Imura T, Chiba R, Yamamoto H, Kawaguchi M, Tsuda Y, Murata K, Yukawa M. 2013. Molecular identification of avian haemosporidia in wild birds and mosquitoes on Tsushima Island, Japan. J Vet Med Sci. 75(3):319–326. doi:[10.1292/jvms.12-0359](https://doi.org/10.1292/jvms.12-0359).

Turcotte A, Bélisle M, Pelletier F, Garant D. 2018. Environmental determinants of haemosporidian parasite prevalence in a declining population of Tree swallows. Parasitology. 145(7):961–970. doi:[10.1017/S0031182017002128](https://doi.org/10.1017/S0031182017002128).

Valkiūnas G, Duc M, Iezhova TA. 2022. Increase of avian Plasmodium circumflexum prevalence, but not of other malaria parasites and related haemosporidians in northern Europe during the past 40 years. Malar J. 21(1):105. doi:[10.1186/s12936-022-04116-7](https://doi.org/10.1186/s12936-022-04116-7).

Van Hemert C, Meixell BW, Smith MM, Handel CM. 2019. Prevalence and diversity of avian blood parasites in a resident northern passerine. Parasite Vectors. 12(1):292. doi:[10.1186/s13071-019-3545-1](https://doi.org/10.1186/s13071-019-3545-1).

Van Rooyen J van, Lalubin F, Glaizot O, Christe P. 2013. Altitudinal variation in haemosporidian parasite distribution in great tit populations. Parasit Vectors. 6(1):139. doi:[10.1186/1756-3305-6-139](https://doi.org/10.1186/1756-3305-6-139).

Waldenström J, Bensch S, Kiboi S, Hasselquist D, Ottosson U. 2002. Cross-species infection of blood parasites between resident and migratory songbirds in Africa. Mol Ecol. 11(8):1545–1554. doi:[10.1046/j.1365-294x.2002.01523.x](https://doi.org/10.1046/j.1365-294x.2002.01523.x).

Walther EL, Carlson JS, Cornel A, Morris BK, Sehgal RNM. 2016. First molecular study of prevalence and diversity of avian haemosporidia in a Central California songbird community. J Ornithol. 157(2):549–564. doi:[10.1007/s10336-015-1301-7](https://doi.org/10.1007/s10336-015-1301-7).

Wilkinson LC, Handel CM, Van Hemert C, Loiseau C, Sehgal RNM. 2016. Avian malaria in a boreal resident species: long-term temporal variability, and increased prevalence in birds with avian keratin disorder. Int J Parasitol. 46(4):281–290. doi:[10.1016/j.ijpara.2015.12.008](https://doi.org/10.1016/j.ijpara.2015.12.008).

Wood MJ, Cosgrove CL, Wilkin TA, Knowles SCL, Day KP, Sheldon BC. 2007. Within-population variation in prevalence and lineage distribution of avian malaria in blue tits, Cyanistes caeruleus. Mol Ecol. 16(15):3263–3273. doi:[10.1111/j.1365-294X.2007.03362.x](https://doi.org/10.1111/j.1365-294X.2007.03362.x).

Yohannes E, Križanauskienė A, Valcu M, Bensch S, Kempenaers B. 2009. Prevalence of malaria and related haemosporidian parasites in two shorebird species with different winter habitat distribution. J Ornithol. 150(1):287–291. doi:[10.1007/s10336-008-0349-z](https://doi.org/10.1007/s10336-008-0349-z).

Yusupova DA, Schumm YR, Sokolov AA, Quillfeldt P. 2023. Haemosporidian blood parasites of passerine birds in north-western Siberia. Polar Biol. 46(6):497–511. doi:[10.1007/s00300-023-03130-y](https://doi.org/10.1007/s00300-023-03130-y).
