# Supplementary material for: Molecular detection of avian parasites in Australian mosquitoes (Culicidae)
Source: J Med Entomol. 2025 Oct 7;62(6):1599–610. doi: 10.1093/jme/tjaf142 (PMC12616232; doi:10.1093/jme/tjaf142)
Supplement: tjaf142_Supplementary_Data [file tjaf142_supplementary_data.zip › Peck_SuppFigure1_NEW.pptx]

## Slide 1
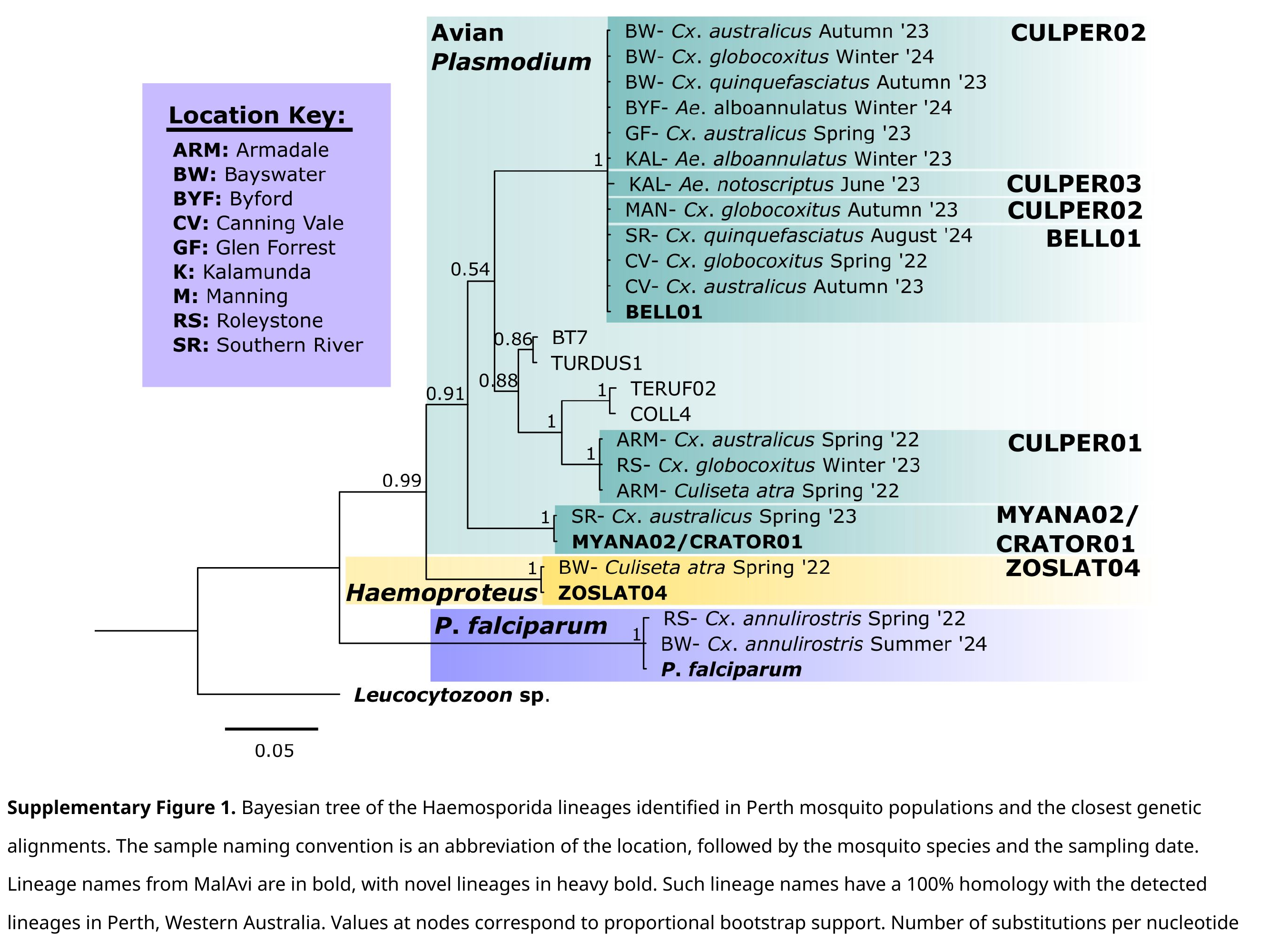

Supplementary Figure 1. Bayesian tree of the Haemosporida lineages identified in Perth mosquito populations and the closest genetic alignments. The sample naming convention is an abbreviation of the location, followed by the mosquito species and the sampling date. Lineage names from MalAvi are in bold, with novel lineages in heavy bold. Such lineage names have a 100% homology with the detected lineages in Perth, Western Australia. Values at nodes correspond to proportional bootstrap support. Number of substitutions per nucleotide position represented by the scale-bar.
