# Supplementary material for: Molecular detection of avian parasites in Australian mosquitoes (Culicidae)
Source: J Med Entomol. 2025 Oct 7;62(6):1599–610. doi: 10.1093/jme/tjaf142 (PMC12616232; doi:10.1093/jme/tjaf142)
Supplement: tjaf142_Supplementary_Data [file tjaf142_supplementary_data.zip › Peck_SuppTable1.docx]

**Supplementary Table 1**. Locations sampled in the present study with site descriptions and coordinates.

| **Location** | **Site description** | **Latitude** | **Longitude** |
| --- | --- | --- | --- |
| Armadale | Border of an urban area and Bungendore Park (large bushland reserve) | -32.170414 | 116.018663 |
| Bayswater | Eric Singleton Bird Sanctuary, artificial wetland located adjacent to Swan River with concentrated wild bird populations | -31.926144 | 115.921381 |
| Byford | Public urban park | -32.226447 | 116.013803 |
| Canning | Kent Street Weir Park, public park | -32.017709 | 115.920022 |
| Canning Vale | Border of a kennel zone and Jandakot Regional Park | -32.097844 | 115.906639 |
| Glen Forrest | Harold Street Reserve, public reserve | -31.911829 | 116.098904 |
| Kalamunda | Jorgenson Park, a public park | -31.972365 | 116.067596 |
| Manning | Lake Gillon at George Burnett , public park. | -32.010901 | 115.879782 |
| Roleystone | Alice Reserve, public reserve | -32.116861 | 116.071141 |
| Southern River | A Kennel Zone area within the City of Gosnells for all dog-related activities, including breeding, boarding and keeping of more than two dogs | -32.117570 | 115.956911 |
